# Structural Design of Ocean Temperature and Depth Sensor with Quick Response and High Sensitivity

**DOI:** 10.3390/s22207756

**Published:** 2022-10-13

**Authors:** Zhaoyue Liu, Yuanchong Zhang, Lina Zeng, Zaijin Li, Hao Chen, Zhongliang Qiao, Yi Qu, Guojun Liu, Lin Li

**Affiliations:** Key Laboratory of Laser Technology and Optoelectronic Functional Materials of Hainan Province, College of Physics and Eletronic Engineering, Hainan Normal University, Haikou 571158, China

**Keywords:** FBG, temperature and depth sensing, elastic diaphragm, liquid filling

## Abstract

The electrical sensing elements used in the traditional XBT (Expendable Bathythermograph) have problems such as low sensitivity and slow response time, and it is difficult to overcome the complex marine environment using the time–depth formula. In this paper, an ocean temperature depth sensor based on brass diaphragm and liquid filling is designed. The stress response time of FBGs with different lengths and the heat transfer time of different liquid materials are compared, and it is found that a fast response of 51 ms can be obtained by using GaInSn liquid for temperature sensing. The center deflection changes of brass diaphragms with different radii are analyzed, and the brass diaphragms with radius and thickness of 10 mm and 1 mm are selected, which still have good elastic properties under the pressure of 5 MPa. The influence of the inner metal shell section radius on the temperature and depth sensitivity is analyzed. When the final section radius is 3 mm, the temperature sensitivity of the sensor is 1.065 nm/°C, the pressure sensitivity is 1.245 nm/MPa, and the response time of temperature and depth is relatively close. Compared with the traditional temperature and depth sensors using empirical formulas for calculation, the data accuracy is improved, and a wide range of sensitivity can be tuned by adjusting the size of the internal metal shell, which can meet the needs of ocean temperature and depth data detection with high sensitivity and fast response time.

## 1. Introduction

The temperature and depth of seawater are the most basic physical quantities of the ocean, and have played an important role in marine scientific research, resource development, meteorological forecasting, military defense, and other fields [1,2]. For a long time, the most commonly used sensors in the world for measuring ocean temperature and depth data have been CTD (Conductivity, Temperature, and Depth) and XBT (Expendable Bathythermograph). Both of these sensors can form profile data of the temperature and depth of the ocean within a certain range. XBT does not need to use large equipment such as winches during use, and can obtain temperature and depth profile data in a small range of sea areas during the process of sailing. XBT is an inexpensive disposable device, and now it is an effective means for countries to quickly measure temperature and depth data [3,4,5].

The temperature measuring elements used in traditional XBT include platinum resistance, thermistors, and other devices, but there are corresponding problems such as low sensitivity, slow response time, and poor thermal stability. At present, for sensors using a thermistor as the sensing element, the response time can generally reach ~100 ms, and relatively high-end products such as Sea-Bird series sensors can reach ~65 ms [6]. Some products do not have pressure-measuring components, but use the time–depth empirical formula to calculate the ocean depth, making it difficult to overcome the complex marine underwater environment [7,8]. As an optical passive device, FBG (Fiber Bragg Grating) has the characteristics of strong anti-interference ability, small size, pressure resistance, and corrosion resistance, and has been used by more and more researchers in ocean temperature and depth sensors [9,10,11]. Due to the low temperature and pressure sensitivity of bare FBGs, many researchers have used different methods to sensitize fibers. In terms of temperature sensing, practical methods include coating the surface or cutting surface of the optical fiber with a temperature-sensitive film [12,13,14,15]; for example, PDMS and SU-8 film can be coated on an Au film based on the plasmon resonance effect [16], with a temperature sensitivity of −1.802 nm/°C, through hydrogen ion-doping of the fiber, filling the pores with SU-8 and alcohol, and special process modifications [17,18,19] to markedly improve the temperature sensitivity of the device. For example, the MZI structure can be prepared on the fiber by the reverse taper process [20] and the OMC can be prepared by fusion and drawing of two traditional communication fibers [21], where the temperature sensitivity can reach 2.326 nm/°C. In terms of depth sensing, most of them operate by amplifying the external stress on the optical fiber; for example, pressure sensitization can be performed through structures such as diaphragms of different materials, cantilevers, and cylinders [22,23,24,25,26,27]. The shipborne consumable temperature and depth sensor based on a beryllium bronze metal diaphragm has a sensitivity of 3.0047 nm/MPa under a pressure of 0–0.6 MPa [28]. There is also an FBG placed in the groove of the thin-walled cylinder, and a pressure sensitivity of 1.198 nm/MPa can be obtained through the deformation of the thin-walled cylinder to sensitize the FBG extrusion [29]. In terms of response time, Tiegen Liu et al. measured the temperature by using the displacement of the diaphragm to change the optical path difference based on the thermal expansion of air. The temperature response speed was 1.3 s in the range of 28 °C to 100 °C [30]. V.V.S.Ch. Swamy et al. placed an FBG on a stainless steel circular diaphragm, and measured the external pressure through the deformation of the diaphragm. The experiment found that the device has a pressure sensitivity of 24.95 nm/MPa, with a pressure-sensing response time of 40 ms [31].

All the above studies use various sensitization methods to improve the temperature and depth sensitivity of the sensor, but there has been less research conducted on the response speed. XBT has been used for rapid measurement of ocean profile data, with a depth measurement range of up to 760 m, for different types of products. The temperature measurement range is −5~35 °C, and the initial falling speed is generally close to 7 m/s. Depending on the marine environment, the falling speed will change greatly. At present, a large number of researchers have been focusing on improving the empirical formula of XBT’s whereabouts [8,32,33], but the temperature profile data obtained by XBT still have deep drift [34]. Still, the faster falling speed requires the sensor to have a faster response time. Whether the sensing response times of the temperature and depth data match or not also affects the accuracy of the profile results. In this regard, designing a temperature and depth sensor structure that can have high temperature and depth sensitivity and fast response speed whilst also maintaining a similar sensing response time for temperature and depth data is the focus of this paper.

In this paper, an ocean temperature and depth sensor based on a brass diaphragm and a liquid-filled structure is proposed, which can realize ocean temperature and depth data sensing with high sensitivity and high response speed. The sensor is mainly composed of an inner metal shell filled with a high-thermal conductivity liquid, an outer metal shell, and a diaphragm for measuring external pressure. Three FBGs are connected in series in an optical fiber, FBG1 is in direct contact with the filling liquid, and the temperature is sensed based on the stress of thermal expansion of the liquid. FBG2 is directly subjected to the stress of the diaphragm through the internal metal shell to sense the pressure, and FBG3 is used for temperature compensation. Compared with the former studies, our design can ensure higher sensitivity and faster response speed, and the response time difference between temperature and depth is shorter, which reduces the error of temperature and depth profile data to a certain extent. The sensor can tune the temperature and depth sensitivity in a wide range by adjusting the size of the inner metal shell and the volume of the filling liquid.

## 2. Structural Design and Principle

**Figure 1:**
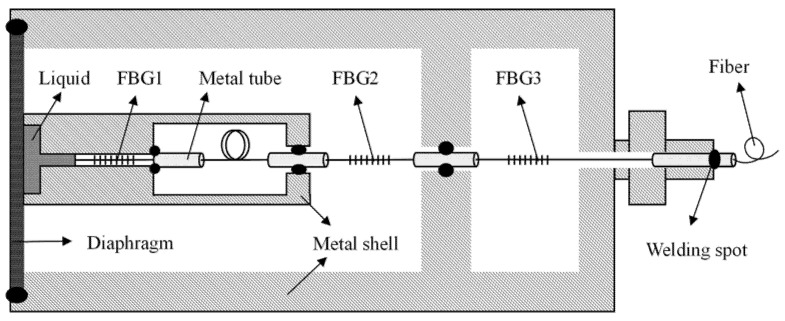
Schematic diagram of temperature- and depth-sensing structure.

The proposed temperature- and depth-sensing structure is shown in Figure 1. This structure consists of an elastic diaphragm, inner and outer metal shells, a sensitizing liquid for improving temperature response, three metal tubes, and three FBGs connected in series. In the temperature measurement part, since the inner metal shell is directly connected to the elastic diaphragm, the volume of the filled sensitizing liquid will change after being affected by the temperature of the diaphragm interface. A metal cylinder similar to a piston is placed between the head end of FBG1 and the liquid. The middle part of FBG1 is not in direct contact with the outer wall, and its tail is fixed in the metal cylinder by epoxy glue and sealed around. The reserved distance of the optical fiber is equal to the distance between the metal cylinder and the metal shell, and the welding is performed by applying pressure to the metal cylinder until it comes into contact with the metal shell. At this time, the optical fiber has been subjected to compressive stress, and the compressive stress on the optical fiber can be adjusted by controlling the reserved distance of the optical fiber. As the volume of the liquid changes, the FBG1 is deformed by the axial stress, so it can measure the external temperature change. In the depth measurement part, the elastic diaphragm is deformed under the influence of external seawater pressure; the internal metal shell and its internal structure can be regarded as a whole, and the displacement of the center of the elastic diaphragm can be regarded as acting directly on FBG2 through the middle metal tube. The tail end of FBG2 is fixed in the metal cylinder, which is deformed under the action of axial stress, so it can measure the external depth change. Between the two metal cylinders (one at the end of FBG1 and the other at the head of FBG2), a bent optical fiber in the middle is used to eliminate the stress intersection between FBG1 and FBG2, so that all the compressive stress acts on FBG2. FBG3 is not affected by pressure and is used to measure the internal temperature of the metal shell. Only the head of FBG1 is in contact with the liquid. The temperature effect of the internal metal shell where it is located is almost the same as that of FBG2 and FBG3, so the three can be regarded as being at the same temperature. The placement of FBG3 realizes temperature compensation, and finally, the optical fiber is led out by the pigtail protection tube.

### 2.1. Principle of Temperature Compensation

According to the above, for FBG1 and FBG2, the metal shells surrounding FBG1 and FBG2 are exposed to the same environment, so in the following, analysis is made taking FBG2 as an example. For FBG2, since it is affected by both external pressure and ambient temperature, the Bragg wavelength shift affected by both strain ε and temperature Δ*T*_2_ changes can be expressed as [35,36]

(1) $$\frac{\Delta \lambda_{B2}}{\lambda_{B2}}=\left(1-P_{e}\right)\varepsilon +\left(\alpha +\xi \right)\Delta T_{2}$$

where *P_e_*, *α*, *ξ* are the effective photoelastic coefficient, thermal expansion coefficient, and thermo-optic coefficient of the fiber, respectively. Since FBG3 is only sensitive to temperature, the Bragg wavelength shift can be written as

(2) $$\frac{\Delta \lambda_{B3}}{\lambda_{B3}}=\left(\alpha +\xi \right)\Delta T_{3}$$

Since these two FBGs are in the same temperature environment, it is assumed that they are subjected to the same temperature change, i.e., Δ*T*_1_ = Δ*T*_2_ = *T*. Equations (1) and (2) can be simplified to

(3) $$\Delta \lambda_{B2}=S\Delta P+S_{T2}\Delta T;\Delta \lambda_{B3}=S_{T3}\Delta T$$

where *S* and *S_T_*_2_ are the pressure sensitivity and temperature sensitivity of FBG1, respectively. *S_T_*_3_ represents the temperature sensitivity of FBG2. Therefore, by equation substitution, we obtain

(4) $$\Delta \lambda_{B2}=S\Delta P+\frac{S_{T2}\Delta \lambda_{B3}}{ST_{3}}$$

Similarly, for FBG1, we obtain

(5) $$\Delta \lambda_{B1}=S\Delta P+\frac{S_{T1}\Delta \lambda_{B3}}{ST_{3}}$$

It can be seen from Equations (4) and (5) that the temperature-induced pressure measurement error is eliminated and temperature compensation is achieved.

### 2.2. Principle of Temperature Sensing

**Figure 2:**
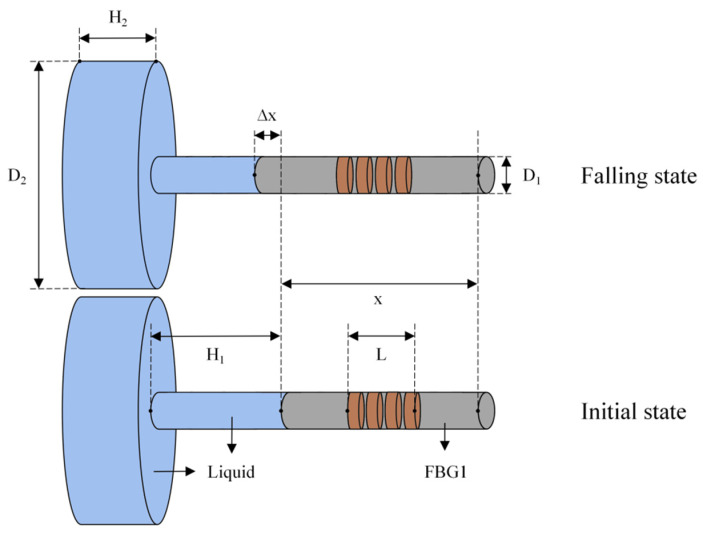
Schematic diagram of the deformation of the liquid column and FBG1 during the falling process.

In the temperature-sensing part, the FBG1 of the temperature and depth sensor is analyzed during the ocean falling process, and the schematic diagram of the deformation of the internal liquid column and FBG1 is shown in Figure 2.

In Figure 2, where *D*_1_ and *D*_2_ are the cross-sectional diameters of the small and large liquid columns in the initial state, respectively; *H*_1_ and *H*_2_ are the heights of the small and large liquid columns in the initial state, respectively; L is the length of the gate region of FBG1; and *x* is the effective length of FBG1 participating in the deformation. During the falling process of the sensor in the ocean, the liquid inside is affected by the external temperature, and its volume decreases. Due to the prestressing of FBG1 in advance, the end is fixed in the metal cylinder with epoxy glue to ensure that FBG1 extends Δ*x* axially as the liquid volume changes. For FBG1, since the elastic deformation of the externally encapsulated metal shell is extremely small, ignoring the influence of the deformation of the metal shell, the elongation of the effective length Δ*x* of FBG1 can be obtained according to the volume change of the liquid.

(6) $$\frac{\Delta x}{\Delta T}=\frac{\alpha_{1}\left(D_{1}^{2}H_{1}+D_{2}^{2}H_{2}\right)}{D_{1}^{2}}$$

where Δ*T* is the temperature change and *α*_1_ is the expansion coefficient of the liquid. According to the change of the gate length of FBG1, the strain of FBG1 can be calculated at every 1 °C as

(7) $$\Delta \varepsilon_{1}=\frac{\alpha_{1}\left(D_{1}^{2}H_{1}+D_{2}^{2}H_{2}\right)}{x\cdot D_{1}^{2}}$$

Then, the wavelength drift of FBG1 is [37]

(8) $$\Delta \lambda_{1}=\lambda_{1}\left(1-P_{e}\right)\Delta \varepsilon_{1}$$

Thus, the temperature sensitivity of FBG1 is

(9) $$K_{T}=\frac{\lambda_{1}\alpha_{1}\left(D_{1}^{2}H_{1}+D_{2}^{2}H_{2}\right)\cdot \left(1-P_{e}\right)}{x\cdot D_{1}^{2}}$$

According to Equation (10), for the same liquid, it is obvious that when the effective length of FBG1 is smaller and the diameter difference between the two liquid columns is larger, the temperature sensitivity of FBG1 is higher.

### 2.3. Principle of Depth Sensing

**Figure 3:**
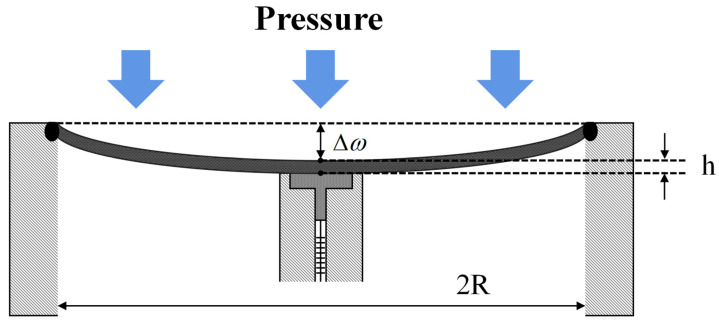
Schematic diagram of diaphragm compression deformation.

In the depth-sensing part, an elastic diaphragm is used as the seawater pressure load. The diaphragm of the sensor is deformed during the falling process, and FBG2 is deformed in the axial direction by the pressure of the metal cylinder. The schematic diagram is shown in Figure 3.

Here, the radius of the diaphragm is *R*, the thickness is *h*, and the displacement of the center affected by the external pressure is Δ*ω*. In practice, the size of the inner metal shell is smaller than that of the diaphragm, and in order to reduce the elastic hysteresis effect of the diaphragm, its amount of deformation needs to be less than one-third of the thickness of the diaphragm. Therefore, ignoring the stress effect of the inner metal shell on the elastic diaphragm, the deformation of FBG2 is equal to the central displacement of the diaphragm [38].

(10) $$\Delta x_{2}=\Delta \omega =\frac{3PR^{4}\left(1-\mu^{2}\right)}{16Eh^{3}}.$$

where *μ* is Poisson’s ratio of the diaphragm material, and *E* is Young’s modulus of the diaphragm material. It can be seen that the deformation of FBG2 changes linearly with the external pressure load, and applying tensile prestress to FBG2 can increase the pressure of the sensor range. Then, the strain of FBG2 is equal to

(11) $$\Delta \varepsilon_{2}=\frac{\Delta \omega }{x_{2}}=\frac{3PR^{4}\left(1-\mu^{2}\right)}{16Eh^{3}x_{2}}$$

According to Equation (9), the pressure sensitivity of FBG2 can be obtained as

(12) $$K_{P}=\frac{3R^{4}\lambda_{2}\left(1-\mu^{2}\right)\cdot \left(1-P_{e}\right)}{16Eh^{3}x_{2}}$$

According to Equation (13), for the same diaphragm material, the larger the radius, the smaller the thickness, and the larger the center deflection, the greater the sensitivity of FBG2. When selecting the diaphragm material, in order to make the temperature response of the sensor faster, a diaphragm material and filling liquid with higher thermal conductivity have been selected here.

## 3. Simulation and Analysis

### 3.1. Response Time Analysis

**Figure 4:**
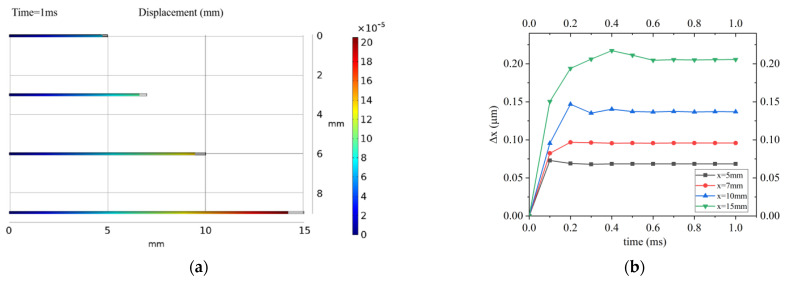
(**a**) Deformation of four FBGs at 1 ms at 1 MPa. (**b**) Deformation and time response of four FBGs at 1 MPa.

The temperature sensing and depth sensing are realized based on the deformation of FBG caused by external pressure. The selection of FBGs with different lengths has a direct impact on the size and response time of the sensor. Fiber gratings with effective lengths of 5 mm, 7 mm, 10 mm, and 15 mm were selected for comparison. Under a pressure of 1 MPa, the response time is in the range from 0 ms to 1 ms, with a step size of 0.1 ms. The deformation amount Δ*x* at 1 ms of these four FBGs and its change with time t are analyzed, and the results are shown in Figure 4.

According to Equation (8), the strain of FBG is independent of its length. The fiber deformation in Figure 4a has been amplified by the same scale. According to Figure 4b, it can be found that these four FBGs are in equilibrium within 1 ms, so the strain response speed of FBG is very small. The response speed of the sensor mainly depends on the speed of temperature transfer and diaphragm deformation. Considering the size of the device and the reflectivity of the center of the FBG, an FBG with an effective length of 10 mm has been selected here.

In the selection of materials, the thermal conductivity of metals is generally better than other materials. At present, for most ocean temperature and depth profile products on the market, the response times are generally within 60 ms, while the response speed of traditional products using platinum resistance as a temperature- and depth-sensing component is generally about 100 ms. The sensor we designed uses copper with high thermal conductivity and good elastic performance as the diaphragm material, and the internal filling liquid also adopts liquid with high thermal conductivity, such as mercury, gallium indium tin, and water. The thermal conductivity and expansion coefficient of the liquids are shown in Table 1 [39].

**Figure 5:**
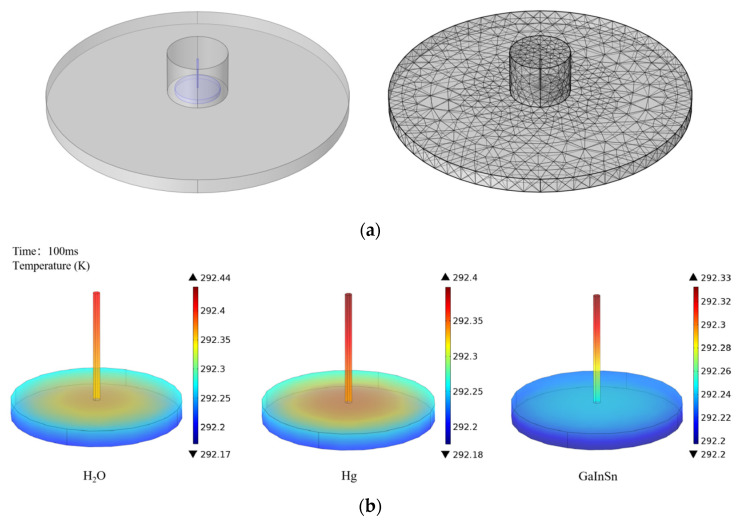
(**a**) Finite element model of diaphragm and filling liquid. (**b**) The heat transfer of the internal liquid at 100 ms when the diaphragm is used as the temperature boundary and the external temperature is 1 K lower than the internal room temperature.

The solidification point of H_2_O is 0 °C, so using H_2_O as a filling liquid affects the measurement range of the sensor. For Hg and GaInSn, the melting points of these two metal liquids are −38 °C and −19 °C, respectively, so the sensor using them can work normally in the marine environment. Simulation and analyses of the heat transfer of these three liquids are performed herein. The default internal liquid column radius is smaller than the external diaphragm radius, the liquid column thickness is 0.3 mm, the filling liquid temperature is set to room temperature (293.15 K), and the external temperature of the diaphragm is 1 K lower than room temperature. The finite element analysis and the heat transfer analysis of the filling liquid are shown in Figure 5.

**Figure 6:**
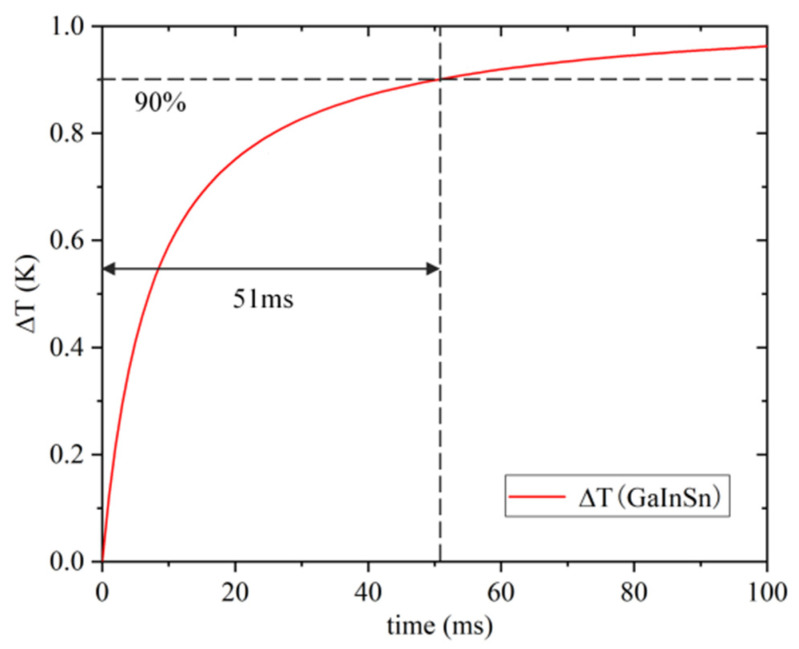
Relationship between temperature change and response time.

In Figure 5b, it can be seen that the bottom liquid column of GaInSn has basically reached the temperature equilibrium state at 100 ms. Compared with water and mercury, the heat conduction speed of GaInSn is faster, and mercury is highly toxic. Furthermore, the use of mercury in the ocean has the hidden danger of leakage and pollution, so we use GaInSn as the sensitizing filling liquid. Taking the volume temperature change of GaInSn liquid reaching 90% as the temperature equilibrium standard, the relationship between temperature and response time can be obtained as shown in Figure 6.

According to Figure 6, using brass as the elastic diaphragm material and GaInSn as the filling liquid material, the internal liquid can reach temperature equilibrium within 51 ms under the temperature difference of 1 °C compared with the outside temperature.

### 3.2. Temperature and Depth Sensitivity Analyses

**Figure 7:**
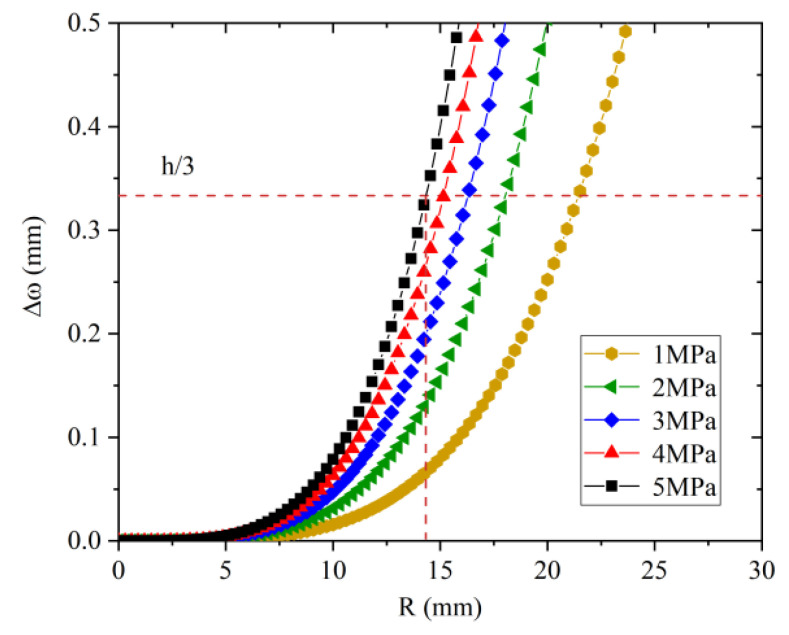
Relationship between the radius of the brass diaphragm and its deflection under different pressure loads.

Since the inner metal shell in the sensor is a cylindrical structure, in order to reduce its influence on the deformation of the diaphragm, a circular diaphragm has been selected here. In the temperature sensing analysis, the thickness of the diaphragm is 1 mm. In order to conform to the theory of small deflection, the radius of the diaphragm and its center deflection are now analyzed. Young’s modulus and Poisson’s ratio of the selected brass diaphragm are 106 GPa and 0.33, respectively. The relationship between the center deflection and the radius is shown in Figure 7.

According to Figure 7, under a pressure load of 5 MPa, brass diaphragms with a thickness of 1 mm and a radius of less than 14 mm satisfy the theory of small deflection, so this sensor uses a brass diaphragm with a radius of 10 mm. In order to ensure good temperature response time and sensitivity, it is necessary to ensure good thermal conductivity between the diaphragm and the liquid, and to also ensure proper inner metal shell size. For the pressure sensitivity of the sensor, since the size of the metal shell directly affects the center displacement of the diaphragm, if the cross-sectional size of the metal shell is large, when the diaphragm is subjected to external pressure, the stress at the welding point of the diaphragm and the metal shell will be reduced. The deformation of the small connection interface reduces the deflection of the diaphragm, so the smaller the size of the inner metal shell, the higher the pressure sensitivity. The pressure sensitivity of the sensor with different internal metal shell section sizes is simulated herein, and the specific parameters of the sensor are shown in Table 2.

**Figure 8:**
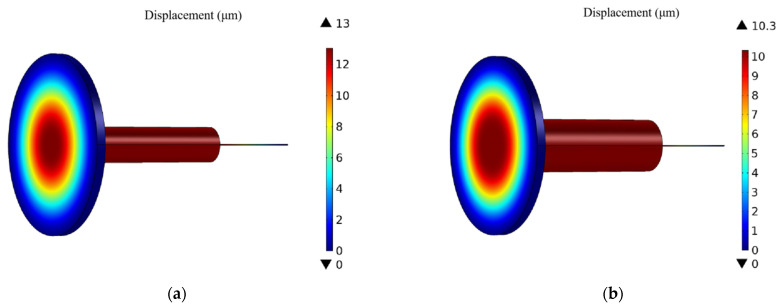
Pressure displacement diagram of the sensor surface. (**a**) The radius of the section is 2 mm. (**b**) The radius of the section is 3 mm.

Using brass metal shells with internal cross-sectional dimensions of 2 mm and 3 mm, and an FBG2 with an effective length of 10 mm, the finite element analysis is carried out herein, and the displacement of the device can be obtained as shown in Figure 8.

It can be seen from Figure 8 that the displacements of the center of the diaphragm are 13 μm and 10.3 μm, respectively. By setting the center wavelength of FBG2 at 1550 nm and substituting into Equations (9) and (12), the pressure sensitivity at this time can be obtained as 1.571 nm/MPa and 1.245 nm/MPa. Obviously, the smaller the cross-sectional radius *R*_in_ of the inner metal shell, the higher the pressure sensitivity of the sensor, and the closer it is to the maximum value. In the modeling process, in order to reduce the deformation of the inner metal shell, the radius of the inner liquid column is set to *R*_L_ = 3*R*_in_/4. In the analysis of the temperature response, the equilibrium state is that the temperature change of the bottom liquid column reaches 90% of the temperature difference of the diaphragm interface. At this time, the temperature of the top liquid column in contact with the sensor and FBG1 has not reached the equilibrium state, and the radius of the top liquid column is small. The contribution of the volume change of the liquid to the deformation of FBG1 is much smaller than that of the bottom liquid column, so the volume change of the top liquid column can be ignored. When calculating the temperature sensitivity of this sensor, Equation (10) is rewritten as

(13) $$K_{T}=\frac{0.9\lambda_{1}\alpha_{1}D_{2}^{2}H_{2}\cdot \left(1-P_{e}\right)}{x\cdot D_{1}^{2}}$$

**Figure 9:**
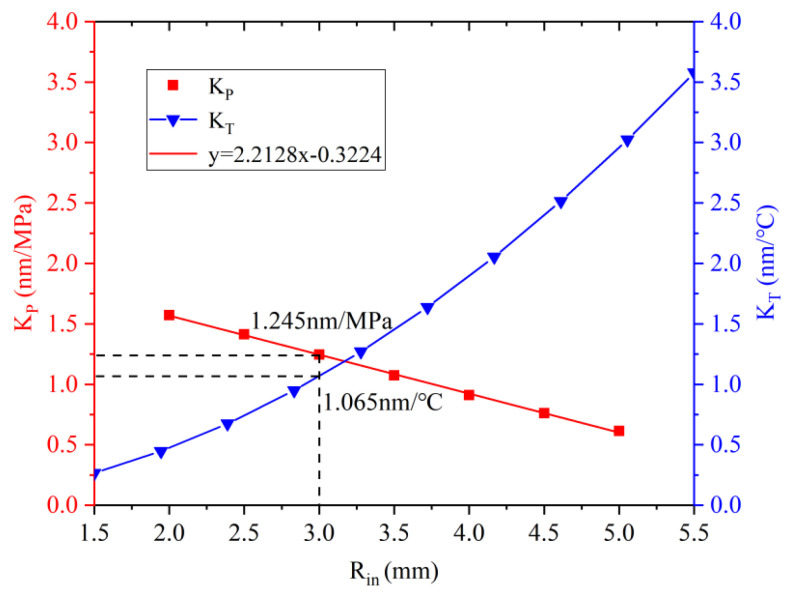
Relationship between temperature depth sensitivity and inner metal shell radius.

The range of the section radius of the above-mentioned internal metal cylinder is set from 2 mm to 5 mm, with a step of 0.5 mm, for analysis. Since the sensor is in the process of falling in the ocean, FBG1 is the recovery process of compressive strain and FBG2 is affected by pressure to generate compressive strain, so the central wavelength of FBG1 is shifted to long wavelength and the central wavelength of FBG2 is shifted to short wavelength. The central wavelength of FBG1 can be set to 1560 nm, and a compressive prestress can be applied. The relationship between the inner metal section shell radius and the temperature depth sensitivity of the sensor is shown in Figure 9.

**Figure 10:**
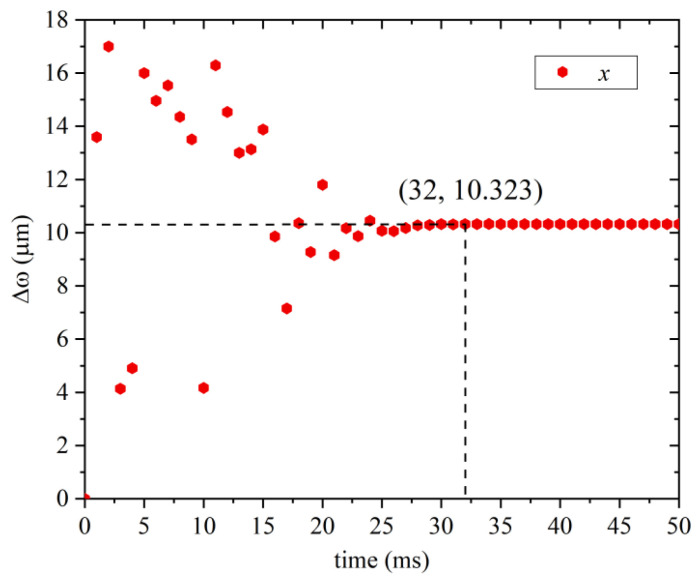
Relationship between diaphragm deformation and response time.

According to Figure 9, as the radius of the inner metal shell increases, the temperature sensitivity of the sensor increases monotonically, and the depth sensitivity decreases linearly. The intersection of the two can ensure that the sensor has high sensitivity in terms of temperature and depth. The value of the temperature and depth sensitivity is 1.1914, the inner metal shell radius of the sensor is selected as 3 mm, the temperature sensitivity is calculated to be 1.065 nm/°C, and the pressure sensitivity is 1.245 nm/MPa. The higher sensitivity range can be tuned by changing the inner metal shell section radius. In terms of the application range of the sensor, according to the analysis of the deflection change of the diaphragm, in order to ensure high sensitivity and small deflection change, the sensor can work in the depth range of 0–200 mm under the condition of applying prestress. During the preparation process of the sensor, the optical fiber and the metal cylinder are encapsulated with epoxy glue and prestressed, and the prestress will be affected by the aging and water expansion of the epoxy glue, resulting in changes in the measurement range and sensitivity of the sensor. This effect will lead to changes in the measurement range and sensitivity of the sensor, so in the process of packaging, the metal shell and three metal tubes need to be sealed. After selecting the appropriate sensor element size, the time response of the diaphragm part used for depth sensing is analyzed herein. Taking 1 ms as a step, the relationship between the diaphragm deformation and the response time in the range from 0 ms to 50 ms is shown in Figure 10.

According to Figure 10, it can be seen that the deformation of the diaphragm gradually stabilizes from the oscillation trend with time until it is in a balanced state at 32 ms, so the depth-sensing response time of this sensor is 32 ms, which is in the same order of magnitude as the temperature-sensing response time (51 ms). Therefore, the sensor can obtain the temperature and depth data completely at 51 ms, which is nearly doubled compared with the traditional XBT, and the response times of temperature and depth are relatively close, which not only ensures high temperature and depth sensitivity, but also improves the temperature and depth matching of profile data accuracy. This is a new method for the detection of ocean temperature and depth profile data.

## 4. Conclusions

In this paper, a temperature and depth sensor based on a diaphragm structure and filling liquid is designed, the response time of this structure is analyzed and simulated, and its temperature and depth sensitivity is calculated. The stress response time of FBGs with different lengths and the heat transfer time of different liquid materials are compared, the center deflection changes of brass diaphragms with different radii are analyzed, and the influence of the inner metal shell section radius on the temperature depth sensitivity is analyzed. Finally, the temperature sensitivity at a radius of 3 mm is 1.065 nm/°C, the pressure sensitivity is 1.245 nm/MPa, the temperature response time is 51 ms, and the pressure response time is 32 ms, which are relatively close. Compared with the traditional temperature and depth sensors using the time–depth empirical formula for calculation, the data accuracy is improved, and it can meet the needs of ocean temperature and depth data detection with high sensitivity and fast response time. The temperature and depth sensitivity of the sensor can be changed by adjusting the radius of the inner metal shell and the volume of the liquid filled. The response time can be optimized by adjusting the materials of the diaphragm and the liquid. Using a liquid with a lower melting point and a more stable thermal expansion coefficient can improve the sensor accuracy and range. In future work, we will manufacture based on the parameters designed above, test the sensor, and carry out comprehensive discussions about the influence of different packaging methods and packaging material characteristics on the time stability of the sensor, so as to ensure that the sensor can work stably in the marine environment.

## Figures and Tables

**Table 1:** Thermal conductivity and thermal expansion coefficients of three liquids.

Liquid (20 °C)	H_2_O	Hg	GaInSn
Thermal conductivity (W/m∙K)	0.599	8.30	16.5
Thermal expansion coefficient (1/K)	2.08 × 10^−^^4^	6.04 × 10^−^^6^	2.5 × 10^−^^5^

**Table 2:** The specific parameters of the sensor.

Parameter	*P*/MPa	*E*/10^5^ MPa	*μ*	*R*/mm	*h*/mm	*λ*/nm	*x*/mm	*H*_2_/mm
Value	1.0	1.06	0.33	10	0.5	1550	10	0.3

## Data Availability

Not applicable.
